# Predicting response to pembrolizumab in metastatic melanoma by a new personalization algorithm

**DOI:** 10.1186/s12967-019-2081-2

**Published:** 2019-10-07

**Authors:** Neta Tsur, Yuri Kogan, Evgenia Avizov-Khodak, Désirée Vaeth, Nils Vogler, Jochen Utikal, Michal Lotem, Zvia Agur

**Affiliations:** 1 Optimata Ltd., Hate’ena St. 10, POB 282, 6099100 Bene-Ataroth, Israel; 2 Institute for Medical BioMathematichs (IMBM), Hate’ena St. 10, 6099100 Bene-Ataroth, Israel; 3 Hadassah Hebrew University Medical Center, Kiryat Hadassah, PO Box 12000, 91120 Jerusalem, Israel; 4 Institute of Clinical Radiology and Nuclear Medicine, University Medical Center Mannheim, Medical Faculty Mannheim, Heidelberg University, Heidelberg, Germany; 5 Medical Faculty Mannheim of Heidelberg University, Theodor-Kutzer-Ufer 1-3, 68167 Mannheim, Germany; 6 German Cancer Research Center (DKFZ), Im Neuenheimer Feld 280, 69120 Heidelberg, Germany; 7 Present Address: Radiology Department, Maccabi Healthcare Services, Yigal Alon Street 96, Tel Aviv, Israel; 8 Present Address: Netzwerk Radiologie, Kantonsspital St. Gallen, Rorschacher Strasse 95, 9007 St. Gallen, Switzerland

**Keywords:** Immunotherapy, Immune checkpoint blocker, PD-1, Pembrolizumab, Prediction algorithm, Effector CD8+ T Lymphocytes, T cell exhaustion, Personalized treatment, Advanced melanoma, Mathematical model

## Abstract

**Background:**

At present, immune checkpoint inhibitors, such as pembrolizumab, are widely used in the therapy of advanced non-resectable melanoma, as they induce more durable responses than other available treatments. However, the overall response rate does not exceed 50% and, considering the high costs and low life expectancy of nonresponding patients, there is a need to select potential responders before therapy. Our aim was to develop a new personalization algorithm which could be beneficial in the clinical setting for predicting time to disease progression under pembrolizumab treatment.

**Methods:**

We developed a simple mathematical model for the interactions of an advanced melanoma tumor with both the immune system and the immunotherapy drug, pembrolizumab. We implemented the model in an algorithm which, in conjunction with clinical pretreatment data, enables prediction of the personal patient response to the drug. To develop the algorithm, we retrospectively collected clinical data of 54 patients with advanced melanoma, who had been treated by pembrolizumab, and correlated personal pretreatment measurements to the mathematical model parameters. Using the algorithm together with the longitudinal tumor burden of each patient, we identified the personal mathematical models, and simulated them to predict the patient’s time to progression. We validated the prediction capacity of the algorithm by the Leave-One-Out cross-validation methodology.

**Results:**

Among the analyzed clinical parameters, the baseline tumor load, the Breslow tumor thickness, and the status of nodular melanoma were significantly correlated with the activation rate of CD8+ T cells and the net tumor growth rate. Using the measurements of these correlates to personalize the mathematical model, we predicted the time to progression of individual patients (Cohen’s κ = 0.489). Comparison of the predicted and the clinical time to progression in patients progressing during the follow-up period showed moderate accuracy (R^2^ = 0.505).

**Conclusions:**

Our results show for the first time that a relatively simple mathematical mechanistic model, implemented in a personalization algorithm, can be personalized by clinical data, evaluated before immunotherapy onset. The algorithm, currently yielding moderately accurate predictions of individual patients’ response to pembrolizumab, can be improved by training on a larger number of patients. Algorithm validation by an independent clinical dataset will enable its use as a tool for treatment personalization.

## Background

Advanced melanoma is the most deadly skin cancer, with a total of 91,279 new cases, and 9320 deaths, expected in 2018 in the United States alone [1]. While early-detected melanoma is mostly curable [2, 3], advanced metastatic melanoma is life-risking. Over the past 10 years, increased biological understanding and access to innovative therapeutic modalities have transformed advanced melanoma into a new oncological model for treating solid cancers [4]. In particular, immune checkpoint blockers (ICB) have shown a major success in the treatment of advanced melanoma [5, 6]. The monoclonal antibody ipilimumab, blocking the cytotoxic T-lymphocyte antigen 4 (CTLA-4), was the first checkpoint blocker approved for the treatment of advanced melanoma, since it shows an objective response rate of 6–11% [7, 8]. The approval was then followed by the one of pembrolizumab and nivolumab—two monoclonal antibody drugs which block the programmed cell death 1 (PD-1) receptor, and show response rates of 30–40% [9, 10]. More recently, a highly toxic combination of ipilimumab and nivolumab was also approved for the treatment of advanced melanoma, with a resulting response rate of about 60% [11, 12]. But in spite of the relatively high response rate of this treatment combination, PD-1 monotherapy, such as the one with pembrolizumab, still remains a pivotal treatment for patients with advanced melanoma, due to its relatively low toxicity and acceptable response rate. Moreover, results of the phase Ib KEYNOTE-001 trial show that a high proportion of patients with metastatic melanoma, who had achieved complete response on pembrolizumab, maintained their complete response for prolonged durations after treatment discontinuation [13]. As ICBs become widely available, the ability to forecast duration of individual response can be critical. How to predict the patient’s response, and adjust treatment plans accordingly, is a big challenge in the current immunotherapy practice [14].

Response rates would be improved and many treatment complications would be prevented if one could identify good responders already before therapy. Indeed, several biomarkers for response to pembrolizumab have been analyzed and the expression of programmed death-ligand 1 (PD-L1) on tumor and immune cells emerged as an acceptable response predictor [15]. Yet, the significant fraction of PD-L1-negative patients who benefit from pembrolizumab suggests that PD-L1 cannot serve as a reliable response biomarker, on its own [16]. In another endeavor, response scales were developed, based on several clinical factors, including localization of metastases, various blood measures, age, and gender. These scoring systems enable to stratify the patient cohort according to the overall response rate and the probability to survive a year from treatment initiation [17, 18]. In other studies, certain immune signatures on the tumor tissue [19, 20], and blood [21] were associated with response, as well. However, the utility of these methodologies has yet to be validated [21].

Acknowledging the urgent need of reliable response predictors, mathematical modelers have joined the efforts to develop tools for predicting personal response to immunotherapy [22]. For example, Kogan et al. [23] proposed a general algorithm for personalizing prostate cancer immunotherapy during the treatment for predicting future response. To this end the authors constructed personalized mathematical models and validated their prediction accuracy retrospectively, by accruing data from a clinical trial of prostate cancer vaccine. This was done using a new methodology of iterative real-time *in*-*treatment* evaluation of patient-specific parameters. Another algorithm for predicting response to cancer therapy is put forward in Elishmereni et al. [24], attacking hormonal treatment of patients with prostate cancer. Here too, the authors developed personalized mathematical models, describing the dynamic pattern of Prostate Specific Antigen. By inputting the personal clinical PSA levels during the first months of treatment, the authors created personal models, and predicted correctly the time to biochemical failure under androgen deprivation therapy in 19 out of 21 (90%) patients with hormone-sensitive prostate cancer.

In the above described algorithms, prediction is made possible only by inputting personal clinical measurements collected during the first months of therapy. While this approach may still be of significant benefit in the design of clinical trials or in the clinics [25, 26], most physicians would prefer to forecast the patient’s response to the drug before treatment onset.

This is the primary goal set in the present work: to develop an algorithm which could be of benefit in the current clinical practice. This will be achieved, first and foremost, by predicting the patient response to therapy before its administration, and secondly, by inputting data that are routinely collected in the clinics, e.g., describing disease progression by the sum of diameters (SOD), as prescribed by the Response Evaluation Criteria In Solid Tumors 1.1 (RECIST 1.1). Most importantly, our goal is to generate instructive output information for the physician’s decision-making process, e.g., aligning the prediction of disease progression with its effective confirmation by computed tomography (CT) or magnetic resonance imaging (MRI).

In the core of our computational algorithm lies a mathematical mechanistic model for the interactive dynamics of the disease, the cellular immune arm and the drug. By inputting clinical and molecular measurements of the patient’s parameters before treatment, the algorithm enables to personalize the model and simulate it to predict the time to disease progression (TTP) of the individual patient under pembrolizumab. Such predictions are expected to assist the treating oncologists in planning the therapy program of the patient.

## Methods

In this section we describe the mathematical mechanistic model we have developed, the model personalization method, the clinical data used for model calibration, and their application for the development of the personalization algorithm.

### Mathematical mechanistic model

**Fig. 1:**
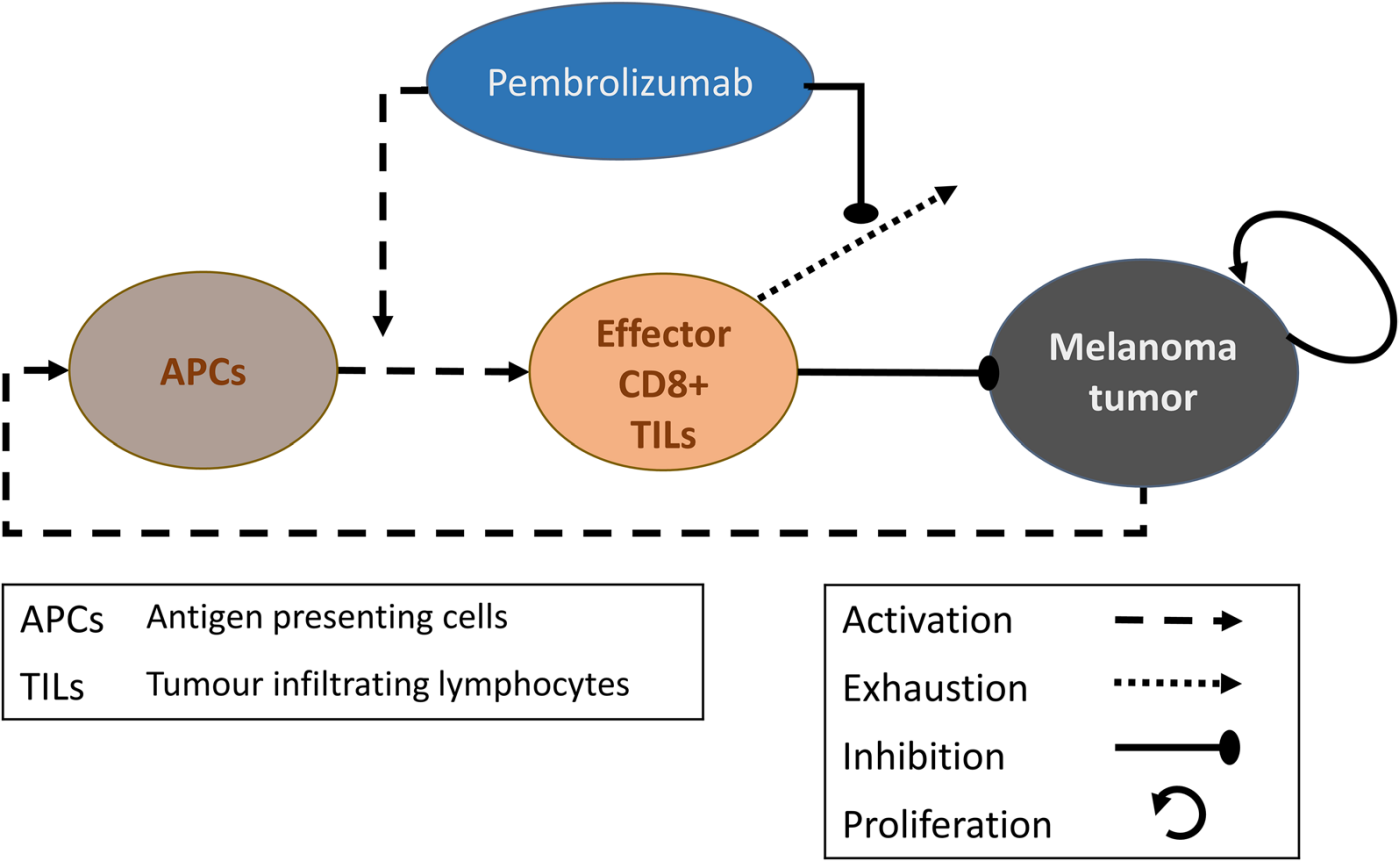
A schematic representation of the model for the main interactions between the melanoma cancer, the cellular immune system, and the immune checkpoint blocker pembrolizumab. The model is based on the following assumptions: tumor cells stimulate antigen-presenting cells, APCs, depending on the tumor immunogenicity; functional APCs activate effector CD8+ T cells, which may eliminate tumor cells; tumor infiltrating lymphocytes, TILs, become exhausted, independently of the tumor cells elimination; tumor growth is determined by its net growth rate and by the rate of its destruction by Effector TILs; immunotherapy extends the activation of effector TILs, and reduces their exhaustion. Annotated ellipses represent the dynamic variables of the model, while arrows represent the interaction between them (see legends in the box)

The mechanistic model we have developed is deliberately simple (*skeletal*), taking into account only the main interactions between the melanoma tumor, the cellular immune system, and the immunotherapeutic drug, pembrolizumab (Fig. 1). Model simplification, incorporating only the bare bones of the system, enables to more easily isolate the effect of each chosen variable and to achieve our stated goal, while retaining the fidelity of description.

The model equations for the dynamics of APCs ($A_{pc}$), T lymphocytes ($T_{il}$) and cancer cells ($M_{el}$) are given below here, while the definitions and estimated values of the model parameters are summarized in Table 1:

1a $$\frac{{dA_{pc} }}{dt} = \alpha_{im} \cdot \frac{{M_{el} }}{{M_{el} + b}} - \mu_{APC} \cdot A_{pc} ,$$

1b $$\frac{{dT_{il} }}{dt} = a_{pem} \cdot \alpha_{eff} \cdot A_{pc} - b_{pem} \cdot \mu_{eff} \cdot T_{il} ,$$

1c $$\frac{{dM_{el} }}{dt} = \gamma_{mel} \cdot M_{el} - \upsilon_{mel} \cdot \frac{{T_{il} \cdot M_{el} }}{{M_{el} + g}}.$$

**Table 1:** Model parameters

Model parameter	Definition	Estimated value	Units	Source
$\alpha_{im}$	Activation rate of APCs	1.161 × 10^3^ to 2.986 × 10^3^	Cells/day	[27, 28]
$b$	Number of tumor cells, required for reaching half of the maximal APC activation rate	3.704 × 10^4^ to 1.476 × 10^5^	Cells	[27–29]
$\mu_{APC}$	Death rate of APCs	0.2310	Day^−1^	[30, 31]
$a_{pem}$	Maximum effect of pembrolizumab on activation of CD8+ T cells	$1 \times 10$ to $1 \times 10^{4}$	–	Model simulations
$\alpha_{eff}$	Activation rate of naïve antigen-specific CD8+ T cells	0.8318	Day^−1^	[32]
$b_{pem}$	Maximum effect of pembrolizumab on the exhaustion rate of CD8+ T cells	0.9	–	Model simulations
$\mu_{eff}$	Death rate of Effector CD8+ T cells	0.1777	Day^−1^	[33]
$\gamma_{mel}$	Net growth rate of tumor cells	0.003269 to 0.08664	Day^−1^	[34]
$\upsilon_{mel}$	Innate death rate of tumor cells by effector CD8+ T cells	0.1245	Day^−1^	[35–37]
$g$	Number of tumor cells, at which the elimination rate reaches half of its maximal value	2.019 × 10^7^ to 1 × 10^8^	Cells	[36, 38]

Numerical analyses and simulations were performed using the ode15s Runge–Kutta ODE solver of Matlab R2016a (The Mathsworks, UK). From the initial time of the simulation (t = 0) to the time of treatment initiation (t = t_1_), the model in Tsur et al. [39] was simulated, and from t_1_ until the end of the simulation period, the model in Eq. (1) was simulated. The effect of pembrolizumab on the immune system and tumor was implemented here by the parameters $a_{pem}$ and $b_{pem}$.

### Patients

The study population included 54 patients with advanced melanoma, who were treated in the past or still receive pembrolizumab as a single-agent between 09/01/2013 and 03/03/2017, at Hadassah Medical Center (HMC; 33 patients), and the University Medical Center Mannheim (UMM, 21 patients). Recruitment to the retrospective clinical trial was subjected to compliance, with the inclusion/exclusion criteria as listed hereafter. Thirty-five of the patients in our dataset did not have a documented progression during their follow-up period and were, therefore, censored, as will be described below.

### Inclusion criteria

1. Gender: female, male.
2. Age: 18 years and older at the start of treatment.
3. Histologically confirmed unresectable Stage III or Stage IV melanoma, as per AJCC staging system.
4. Prior radiotherapy or other oncological treatments must have been completed at least 2 weeks prior to drug administration.
5. Measurable disease by CT, or Positron Emission Tomography CT (PET-CT), or MRI, per Response Evaluation Criteria In Solid Tumors (RECIST 1.1) [40].
6. Patient has at least one quantitative measurement of at least one target lesion (primary tumor or metastasis) before treatment.
7. Patient has at least one quantitative measurement of at least one target lesion (primary tumor or metastasis) during or after the treatment.
8. Patient has at least one recorded visit to the treating oncologist before treatment.
9. Patient has at least one recorded visit to the treating oncologist during or after the treatment.
10. Treatment as per Standard of care for melanoma.

### Exclusion criteria

1. History of another malignancy within the previous 2 years, except for adequately treated Stage I or II cancer currently in complete remission, or any other cancer that has been in complete remission for at least 2 years.
2. Ocular melanoma.

The data collected from the medical records of the patients included demographics, information about the diagnosis and primary tumor, staging, applied oncological treatments, detailed information about administration of pembrolizumab (specific protocols), imaging data and blood measures, including relative lymphocyte counts. Baseline information and follow-up duration of the patients are summarized in Table 2.

**Table 2:** Characteristics of the patient cohort, and baseline information

Age mean (range), years	68.5 (32.5–91.5)
Gender	
Male n (%)	39 (72%)
Female n (%)	15 (28%)
Primary nodular melanoma	
Yes n (%)	11 (20%)
No n (%)	41 (76%)
Undetermined n (%)	2 (4%)
Breslow thickness mean (range), mm	4.11 (0.37–28)
BRAF V600 status	
Wild type n (%)	33 (61%)
Mutant n (%)	13 (24%)
Undetermined n (%)	8 (15%)
Previous ipilimumab n (%)	22 (41%)
Previous nivolumab n (%)	0 (0%)
Baseline tumor size before pembrolizumab mean (range), mm	79 (5–247)
Follow-up period median (range), months	9 (2–35)

### Imaging data

Baseline and follow-up CT and MRI scans were retrospectively reviewed by radiologists at HMC and UMM. The time interval between consecutive scans was around 3 months. In each scan the maximal and perpendicular diameters of each morphologic detectable lesion in the x–y plane were evaluated, using the GE Centricity PACS software of GE Healthcare at HMC, and a dedicated post processing software (Syngo.Via, Siemens Healthineers, Erlangen, Germany) at UMM. We documented the organ each lesion was found in, and noted new lesions appearing in follow-up scans.

### Response evaluation

Response evaluation and identification of target lesions was made based on the RECIST 1.1 guidelines. A target lesion is defined by its size, having a minimum diameter of 10 mm for a non-nodal lesion, or a minimum diameter of 15 mm in case of a lymph node. In accordance with RECIST 1.1, we selected up to two target lesions per organ and a maximum of five in total. We summed up the target lesions diameters to obtain the SOD at each tumor size assessment for every patient. At each time of clinical tumor size evaluation we assigned to the patient one of the RECIST 1.1-defined response types, as specified in [40].

## Development of the personalization algorithm

### Selection of the personal model parameters

In order to personalize our mathematical model we first selected the model parameters which are expected to significantly affect the response and to vary among patients. We chose to personalize, that is, to adjust the values within a certain range, the following two parameters: (i) effect of pembrolizumab on the activation of CD8+ T cells ($a_{pem}$), (ii) tumor growth rate ($\gamma_{mel}$). The choice to personalize these two parameters was based on our theoretical analysis of the mathematical model described in Eq. (1), showing that changes in the maximum effect of pembrolizumab on the activation of CD8+ T cells, $a_{pem}$, affect the balance between tumor growth rate and the efficacy of the immune system. We inferred that this parameter varies among patients. Furthermore, stability analysis of the mathematical model shows that in an untreated host, the net growth rate of tumor cells, $\gamma_{mel}$, is the parameter having the largest effect on the tumor dynamics [39]. For this reason, we consider this parameter as an individual parameter as well.

For personalizing the mathematical model, we set the range of $a_{pem}$ values to allow different tumor dynamics, as a result of the therapy. Moreover, we estimated the range of $\gamma_{mel}$ from the doubling time ($\Delta t$) of human melanoma metastases: $\gamma_{mel} = \frac{{{ \ln }\left( 2 \right)}}{\Delta t}$, which was estimated by Carlson [34] and Joseph et al. [41], as described in detail in Table 1. In order to improve parameter identifiability, we dichotomized $\gamma_{mel}$ to be equal to either the minimum or the median of its range. The ranges of the personalization parameters are summarized in Table 3. For the first iteration of the fitting algorithm we chose the initial guess of each personalization parameter as the median of its range. As mentioned above, all other parameters were fixed to their values reported in Table 1.

**Table 3:** Ranges of the personalization parameters for identifying the patient-specific model parameters

Model parameter	Definition	Personalization parameter range	Units
$a_{pem}$	Maximum effect of pembrolizumab on activation of CD8+ T cells	$10$ to $10^{4}$	–
$\gamma_{mel}$	Tumor growth rate	$3.269 \times 10^{ - 3}$ or $5 \times 10^{ - 3}$	Day^−1^

### Creating the personal models

To fit the model to data from the training set, we minimized the sum of squared errors of the observed and simulated tumor size, using ‘*fmincon*’ function in Matlab. The goodness of fit was determined by calculating the coefficient of determination, R-squared, for the fitted versus clinically measured tumor sizes of all the patients in the dataset. Subsequently, we determined the functions that enable personalization of the mathematical model, by considering several clinically measured factors, whose values were available for the majority of the patients in this study, in at least one time point before treatment onset, or at least in one time point at an early stage of the treatment (Table 4). Some of these factors, including lactate dehydrogenase (LDH) levels, relative counts of blood lymphocytes (LY%), and baseline SOD, are known to be associated with the response to pembrolizumab [17, 42]. The relationships between the other clinical variables considered for the personalization functions, and outcome under pembrolizumab, were examined by correlation analysis. We used four standard statistical methods to analyze the relationships between personal clinical data and model parameters: Pearson coefficient, receiver operating characteristic (ROC) analysis, confusion table and Cohen’s kappa (κ). The obtained relationships were the basis for the formulation of the personalization functions.

**Table 4:** Availability of measurements in the recruited patient dataset

Potential covariate	Availability (number of patients, out of 54)
Age	54
Breslow thickness	43
LDH	51
LY%	53
SOD at the baseline^a^	54
Cutaneous malignant melanoma (Y/N)	52
Nodular melanoma (Y/N)	52
BRAF V600 mutation (Y/N)	46

Measurements of parameters that are potential covariates for determination of the personal models (Y/N refers to a test outcome)

^a^Sum of diameters of measurable lesions at the baseline, as defined by RECIST 1.1 [40]

To overcome variations in the clinical and molecular values that are due to differences in the measurement and calibration techniques, used in each medical center, we normalized each measured value ($X$), relative to its given range, between $X_{min}$ and $X_{max}$, as specified for each of the two subsets. The normalized covariate value ($\hat{X}$) is

2 $$\hat{X} = \frac{{X - X_{min} }}{{X_{max} - X_{min} }} .$$

From the individual model fits we obtained the personal model parameters for all patients in the training set. From the clinical record files of each patient we retrieved the relevant clinical measurements for all patients at baseline, and around the time of the first follow-up imaging assessment. For estimation of $a_{pem}$ from the clinical/molecular measurements we used the k-Nearest Neighbors (k-NN) algorithm. The number of nearest neighbors, k, was taken to be the integer part of the square root of the total number of patients (N = 54), i.e., k = 7. In case of missing data of a clinical/molecular measurements we replaced the missing value by the average value for this clinical factor, obtained from the data of the rest of the patients. Missing values of binary clinical/molecular factors were set to 0. We validated the resulting personalization functions by the Leave-One-Out cross validation (LOO CV) method. In order to evaluate the personal $\gamma_{mel}$ values from the clinical measurements, we trained a classification tree, using the LOO CV, as above. After predicting the parameter values of each patient we simulated the personalized models (using the ode15s solver of Matlab), derived the simulated tumor size at the days of the imaging assessments, and evaluated TTP based on RECIST 1.1 [40].

### Analysis of the TTP results

To evaluate the quality of TTP prediction, we compared the predicted versus the clinically observed TTP in three time intervals, including 0–90 days, 90–150 days and 150–365 days, from pembrolizumab initiation. We also took into account the number of patients for whom no disease progression was indicated during their follow-up period. As was mentioned above, from the practical point of view, the resolution of TTP predictions should be as coarse as the planned CT/MRI scanning schedule.

We categorized our predictions according to these time intervals, and generated a confusion table. To calculate the corresponding value of the Cohen’s kappa (κ), we applied the multidimensional formula of Warrens [43], who defines the proportion $p_{1}$ of patients, whose simulated time interval of the TTP ($t_{s}$) matched the reference one ($t_{r}$). The proportion $p_{1}$ is the ratio between the number of these patients, denoted $N_{TTP} \left( {t_{s} = t_{r} } \right)$, and the total number of patients in the cohort ($N = 54$):

3 $$p_{1} \equiv \mathop \sum \limits_{r,s = 1}^{4} \frac{{N_{TTP} \left( {t_{r} = t_{s} } \right)}}{N}.$$

The proportion of patients in each time interval is denoted $p_{2}$. It is calculated from the number of simulated, and observed disease progression incidences in each time interval, denoted $N_{TTP} \left( {t_{s} } \right)$, and $N_{TTP} \left( {t_{r} } \right)$, respectively:

4 $$p_{2} \equiv \mathop \sum \limits_{{t_{s} ,t_{r} = 1}}^{4} \frac{{N_{TTP} \left( {t_{s} } \right)}}{N} \cdot \frac{{N_{TTP} \left( {t_{r} } \right)}}{N}.$$

The multidimensional Cohen’s kappa ($\kappa$) is

5 $$\kappa = \frac{{p_{1} - p_{2} }}{{1 - p_{2} }}.$$

The data in the confusion table can be categorized into six different outcomes, as follows:

1. Progressive disease was clinically evidenced by imaging assessments, as well as predicted by the algorithm, at the same time interval ($t_{r} = t_{s}$).
2. The algorithm’s simulated TTP was predicted to precede the observed TTP ($t_{s} < t_{r}$).
3. The algorithm’s simulated TTP was predicted to occur later than the observed TTP ($t_{s} > t_{r}$).
4. Progressive disease was not clinically observed, but was predicted by the algorithm ($t_{r} = 4;t_{s} = 1,2,3$).
5. Progressive disease was clinically observed, but was not predicted by the algorithm ($t_{r} = 1,2,3;t_{s} = 4$).
6. Progressive disease was neither observed nor predicted by the algorithm, during the follow-up period ($t_{r} = t_{s} = 4$).

## Results

This section is divided into two parts. The first part describes the personalization algorithm and its development, while the second part shows the predictions of the personal TTP of the patients, obtained by using the personalization algorithm.

### The personalization algorithm

First, we outline the personalization algorithm we have developed for predicting response to pembrolizumab in a patient with advanced melanoma:

1. Input personal baseline data.
  - Tumor burden from imaging scans.
  - Primary tumor information.
2. Construct a personalized model by inputting the personal clinical data in the algorithm’s personalization functions and calculating the values of the personal parameters.
3. Input the calculated personal parameters in the personal model.
4. Simulate the personal model and extract the predicted tumor size, periodically, at predetermined time intervals, for example, every 3 months.
5. Determine disease state for each predicted tumor size, in conjunction with previously predicted tumor sizes and RECIST 1.1 criteria.
6. Determine the personal timing of progressive disease and the personal TTP.

### Algorithm development: retrieving personal model parameters and evaluating TTP in the training set

The development of the above algorithm is described hereafter.

**Fig. 2:**
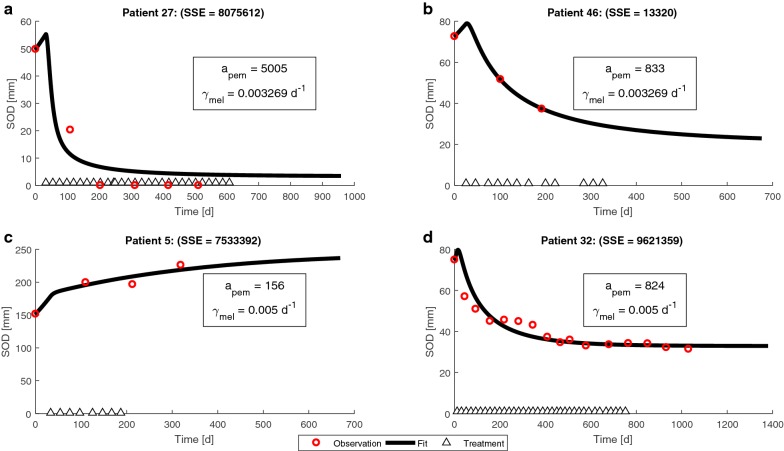
Representative fitting results of patients, whose target lesions completely shrunk under treatment with pembrolizumab (**a**), shrunk by more than 30% from baseline size (**b**), increased by over 20%, relative to the nadir measurement (**c**), was stabilized, as determined when the conditions for disease progression, partial response, and complete response were not met (**d**). The ranges of the personalization parameters used for the simulation are specified in Table 3. *SOD* sum of diameters

In the first stage of algorithm development we verified that the clinical information we have is sufficient for the training of the algorithm. We found that all four response categories of RECIST 1.1 are represented in the collected clinical information of our patient cohort, during the follow-up period: (i) full response of the target lesions (e.g., Fig. 2a); (ii) shrinkage of the target lesions by more than 30% from baseline size (e.g., Fig. 2b); (iii) progression of the target lesions, indicated by an increase of ≥ 20% relative to the nadir (e.g., Fig. 2c); (iv) stability in the size of the target lesions, not meeting the aforementioned conditions of shrinkage or progression (e.g., Fig. 2d). This information ensures that the training of the algorithm will be comprehensive. In the next stage we employed the longitudinal tumor size evaluations for retrieving the personal model parameters. This was done by fitting the model to the SOD time series, calculated from the clinical data (Fig. 2).

**Fig. 3:**
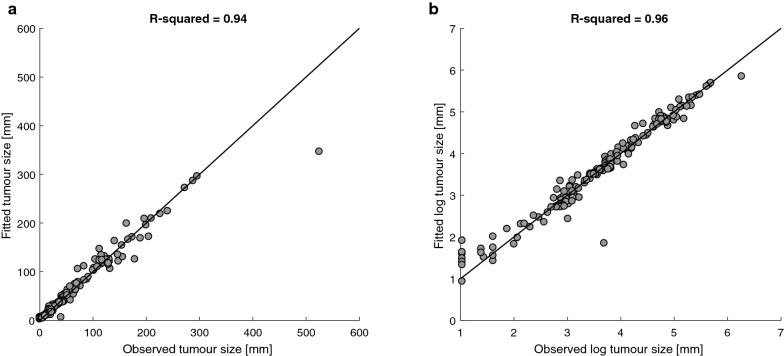
Fitting results of the model-simulated tumor size in the patients’ cohort (N = 54), with the clinically observed tumor sizes. **a** Each point shows the fitted versus the clinically measured sum of diameters (SOD) of a patient, at the time an imaging assessment took place in the clinic. The observed SOD on the reference line equals to the fitted values. The personalization parameter ranges used for the simulation are specified in Table 3, and the values of the other model parameters are summarized in Table 1. Numerical analyses and simulations were performed using the ode15s Runge–Kutta ODE solver of Matlab R2016a (The Mathsworks, UK). From the initial time of the simulation (t = 0) to the time of treatment initiation (t = t_1_), the model in Tsur et al. [39], was simulated, and from t_1_ until the end of the simulation period, the model in Eq. (1) was simulated. The effect of pembrolizumab on the immune system and tumor was implemented here by the parameters ${\text{a}}_{\text{pem}}$ and ${\text{b}}_{\text{pem}}$. **b** Fitted versus observed SOD on a log scale. Values of 0 were excluded from the dataset for calculation of R-squared

In order to estimate the goodness of the fit of the models, we compared between the clinically observed and the fitted tumor sizes of all the patients in the cohort (Fig. 3). Comparison of the absolute and log-scaled sizes yielded R^2^ = 0.94, and R^2^ = 0.96, respectively.

We then compared the TTP derived from the fitting results to the clinically observed TTP, by counting the number of disease progression events in each one of four categories of time intervals, as described in the “Methods” section, and summarized in Table 5.

**Table 5:** Model-simulated versus clinically- measured TTP

Simul. TTP	Clinic. TTP			
	0–90 days	90–150 days	150–365 days	No progressive disease during follow-up
0–90 days	*8* (14.8%)	0 (0%)	2 (3.7%)	0 (0%)
90–150 days	0 (0%)	*3* (5.6%)	0 (0%)	0 (0%)
150–365 days	0 (0%)	0 (0%)	*2* (3.7%)	0 (0%)
No progressive disease during follow-up	2 (3.7%)	1 (1.85%)	1 (1.85%)	*35* (64.8%)

The simulated TTP (Simul.) was obtained by fitting the simulations of the mathematical model in Eq. (1) to the clinical results (Clinic.). The cells in the table include the number of cases and the percentage of the total number of patients in the cohort, (in brackets; *N* = 54), which satisfy one of the six possible outcomes described in the “Methods” section. The italicized numbers represent the number of cases, for which the algorithm correctly predicted whether progression will occur, and correctly predicted the time interval during which progression would occur; Cohen’s *κ* = 0.773

**Fig. 4:**
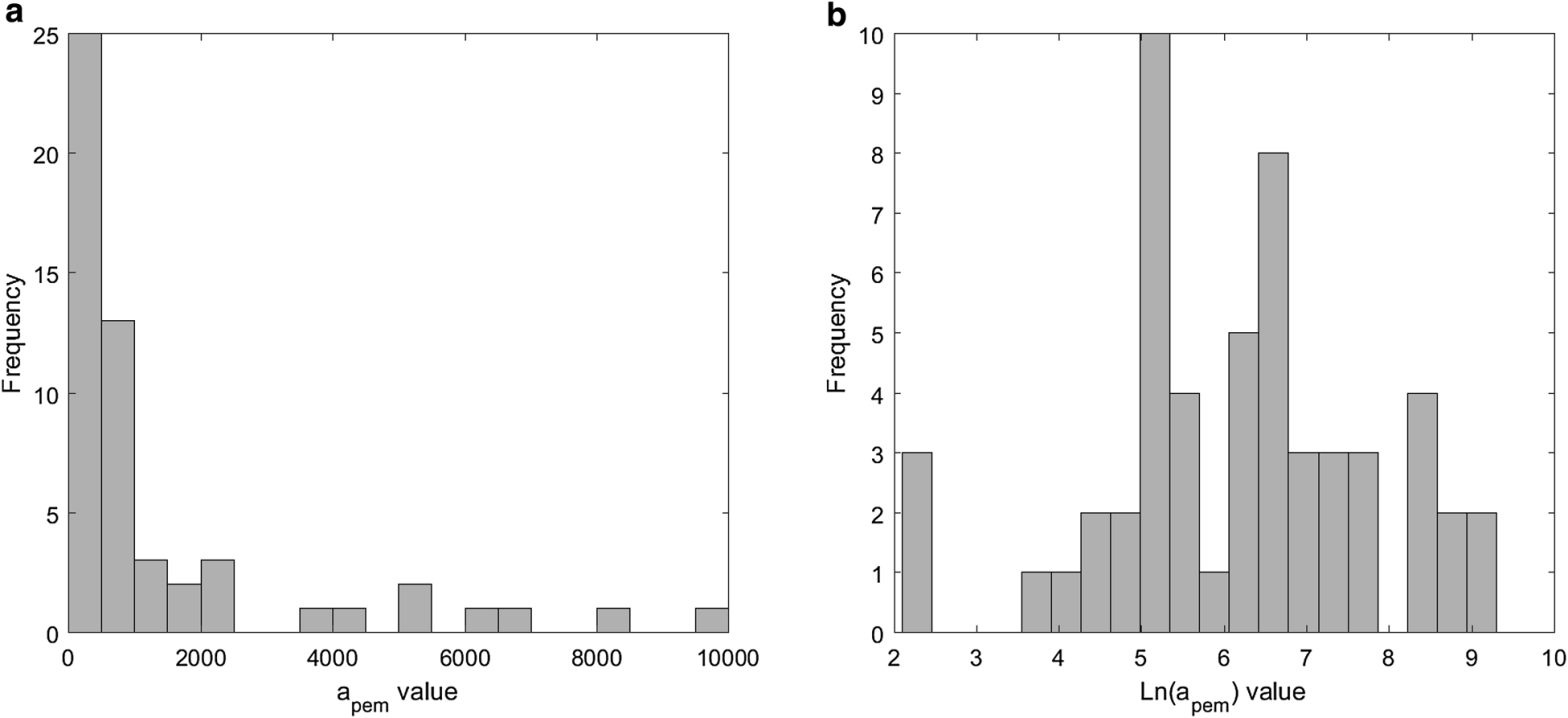
Histogram of $a_{pem}$ values, obtained from fitting of the mathematical model to the clinically observed tumor size. The initial range of $a_{pem}$ for the fit is defined in Table 3. **a** Absolute values of $a_{pem}$. **b** Transformed values of $ln\left( {a_{pem} } \right)$

From the histogram of the fitted $a_{pem}$ values, we learned that the distribution of this parameter in the patient population is approximately log-normal (Fig. 4). This implies that lower values are more frequently encountered than large ones. Thus, in order to reduce the bias in the prediction of this parameter, we applied a logarithmic transformation to the values of $a_{pem}$.

### Predictions of the personal models

As summarized in Tables 6, 7, we found that the value of $a_{pem}$ is most correlative with the baseline SOD (Table 6), and the value of $\gamma_{mel}$ is most correlative with Breslow thickness and the status of nodular melanoma (Table 7).

**Table 6:** Pearson correlations between the values of model parameter $ln\left( {a_{pem} } \right)$ and the clinical personal measures

Potential clinical covariate	Pearson correlation coefficient	Significance (p-value)
Age	0.051	0.77
Breslow thickness	− 0.089	0.53
LDH	− 0.573	9.2 × 10^−6^
LY%	0.370	0.01
SOD at the baseline	*− 0.703*	*3.5 × 10* ^*−9*^

Among the tested clinical measures, the baseline sum of diameters (SOD) was found to be most significantly correlated with $\ln \left( {a_{pem} } \right)$ (in italics)

**Table 7:** (a) Correlations between $\gamma_{mel}$ and clinical measures, with multiple potential values. (b) Correlations between $\gamma_{mel}$ and binary covariates

(a) $\gamma_{mel}$ and clinical measures			(b) $\gamma_{mel}$ and binary covariates	
Biomarker	ROC AUC	Maximal Cohen’s kappa (κ)	Biomarker	Cohen’s kappa (κ)
Age	0.56	0.219	Malignant melanoma status	0.185
Breslow thickness	*0.63*	*0.304*	Nodular melanoma status	0.258
LDH	0.52	0.128	BRAF V600 status	0.124
LY%	0.52	0.156		
SOD at the baseline	0.61	0.251		

The maximal AUC and Cohen’s kappa (*κ*) values, obtained from ROC analysis (in italics) were obtained for Breslow thickness (see “Methods”)

**Fig. 5:**
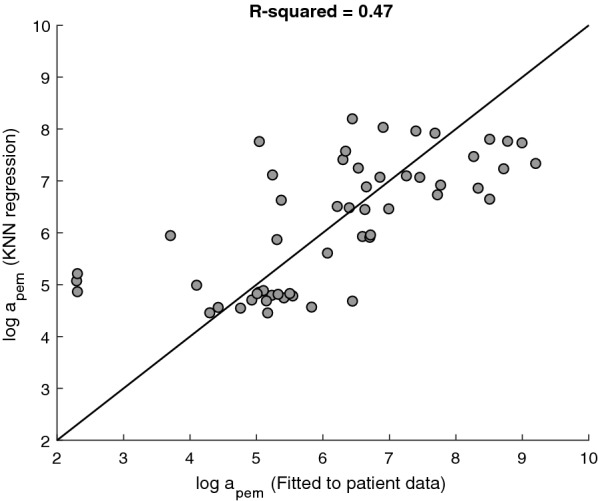
Patient-specific values of $ln\left( {a_{pem} } \right)$, as obtained from fitting the mathematical model to the data of each patient in the training set, versus the estimated values of $ln\left( {a_{pem} } \right)$ by a Leave-One-Out cross-validation (COO CV) of the k-NN algorithm. Each point represents the parameter values of one patient and the reference line satisfies equality between the fitted and regression-derived parameter values (see “Methods” section)

We calculated the R^2^ value of the parameter values derived by the k-NN algorithm versus the fitted ones, in order to estimate the goodness of fit. The value of R^2^ = 0.47 obtained for the baseline SOD, refers to results of LOO CV. The plot of the fitted $ln\left( {a_{pem} } \right)$ value for each patient, versus the k-NN algorithm-derived value is shown in Fig. 5.

Using all the clinical factors listed in Table 4 to train and optimize a classification tree, and validating the classification by LOO CV, we found that the tree which most correctly classified the values of $\gamma_{mel}$ was obtained from the data of Breslow thickness and status of nodular melanoma. The comparison between the classified and fitted values of $\gamma_{mel}$, with the use of these two covariates, are summarized Table 8.

**Table 8:** Confusion table for the classification of the model parameter, $\gamma_{mel}$

$\kappa = 0.222$	Fitted value of $\gamma_{mel}$	
	Value 1	Value 2
Classification-derived value of $\gamma_{mel}$		
Value 1	19	13
Value 2	8	14

The parameter $\gamma_{mel}$ can take on two numerical values, namely 0.005 day^−1^ (value 1), or 0.003269 day^−1^ (value 2). Presented in the table are the number of cases in which value 1 of $\gamma_{mel}$ was fitted, and identified as value 1 by the classification algorithm (true positive; upper-left cell), or value 2 (false negative; lower left cell), and the number of cases in which value 2 of $\gamma_{mel}$ was fitted, as well as classified as value 2 (true negative; right lower cell), or misclassified as value 1 (false positive; right upper cell). The calculated Cohen’s kappa, $\kappa ,$ suggests a fair agreement of predictions to fitting results

Based on the above results we constructed the personalization functions, estimating the values of $a_{pem}$ and $\gamma_{mel}$ of each patient in the validation set, from the baseline SOD for $a_{pem}$, and Breslow tumor thickness, and status of nodular melanoma for $\gamma_{mel}$ (Tables 6, 7). We completed the personalization algorithm by implementing in it the personalization functions.

### Prediction of the TTP using the personalization algorithm

**Fig. 6:**
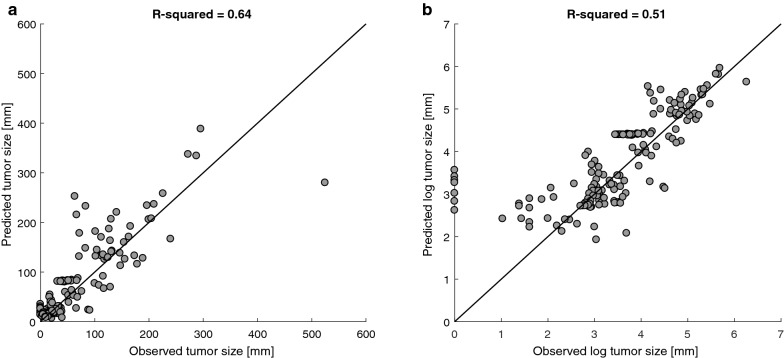
Comparison between the sum of diameters (SOD), derived by the personalization algorithm, and the value measured from imaging assessments, at each clinically measured time point, for all patients, presented on a normal scale (**a**), and on a log scale (**b**). The reference line marks equality between the fitted and predicted SOD values. Values of 0 were excluded from the dataset for calculation of R-squared

We compared the predictions obtained by the personalization algorithm with the clinically measured tumor sizes in all patients. We evaluated the goodness of fit of the algorithm-predicted and clinically-measured tumor size, as shown in Fig. 6.

From the predicted tumor size dynamics, we also predicted the TTP, and compared it to the value estimated according to the clinically assessed progression. Results are shown in Table 9. The evaluation of the Cohen’s kappa, κ = 0.489, suggests a moderate agreement between the prediction and clinical data.

**Table 9:** Personal predictions of TTP

Pred. TTP	Clinic. TTP			
	0–90 days	90–150 days	150–365 days	No progressive disease during follow-up
0–90 days	*6* (11.1%)	0 (0%)	2 (3.7%)	2 (3.7%)
90–150 days	0 (0%)	*2* (3.7%)	0 (0%)	3 (5.6%)
150–365 days	0 (0%)	0 (0%)	*1* (1.8%)	0 (0%)
No progressive disease during follow-up	4 (7.4%)	2 (3.7%)	2 (3.7%)	*30* (55.6%)

Comparison between the TTP derived from model predictions (pred.) of the personalization algorithm, and the clinically measured (clinic.) TTP. Each cell includes the number of cases and percentage from the total number of patients in the cohort (in brackets; *N* = 54), which satisfy one of the six possible outcomes (see “Methods”). The italicized numbers represent the number of cases for which the algorithm correctly predicted whether disease progression will occur, and correctly predicted the time interval in which it occured. Note that our algorithm predicted no progression during the 1-year follow-up period for 30 out of the 35 patients who had not shown clinical progression during that period (bottom right cell).  Cohen’s *κ* = 0.489

**Fig. 7:**
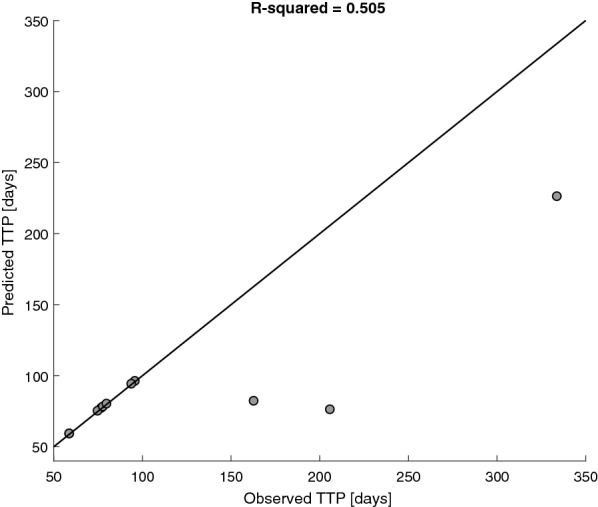
Comparison between the predicted time to progression (TTP) and the measured clinical TTP, including only the cases in which disease progression was determined clinically, as well as by the personalization algorithm. Points on the reference line satisfy equality between the observed and computationally derived TTP

For the patients who had progressive disease according to our personalization algorithm, we compared the predicted TTP to the clinically observed one (Fig. 7). The results show moderate agreement of the predictions with the clinical observations (R^2^ = 0.505).

## Discussion

Treatment with ICB has proven successful, as it produces a significant clinical benefit in a subset of patients. However, identification of the potentially responsive patients before treatment initiation still remains a challenge, and the availability of personal response predictors has been pointed out as an unmet clinical need [44–47]. Here we showed that the personalization algorithm we developed can serve as a virtual response predictor in the clinic, along with clinical information about baseline tumor size, Breslow thickness, and the status of nodular melanoma. Taking into account the low life expectancy of untreated patients with advanced melanoma, and the involved side effects and high immunotherapy costs [48], the ability to pre-select patients for these treatments can significantly improve the quality of life of the patients.

The personalization algorithm we developed enables predictions of the time to progression, as defined by RECIST 1.1. Nowadays, the first response assessment in the clinic takes place at around 3 months into the treatment. As many patients progress within these first 3 months [49–51], the algorithm predicting the TTP before treatment can save several months of administration of an incompatible and expensive drug. For patients who benefit from the treatment, the algorithm provides information on the duration of the response.

Prediction of the type and duration of response is a unique addition of this study to the knowledge gained from previously developed biomarkers for ICB. Several markers in the tumor microenvironment and peripheral blood are associated with response to ICB in patients with malignant melanoma [52]. However, there is no way to quantify the relationships between the biomarker levels and the expected response, as yet. For example, elevation of the baseline LDH level is associated with shorter overall survival (OS) of patients with malignant melanoma under anti-PD-1 treatments [53]. However, the survival time of individual patients cannot be predicted by this marker. In our study, clinical disease progression was observed in all patients who had an elevated LDH level before treatment onset and more than 10% increase of the LDH level on the first CT scan (11 out of 29, 38%). In contrast, disease progression occurred in only 4 out of 18 patients who initially had elevated LDH levels, but less than 10% LDH change from baseline on the first CT scan. Therefore, the change from baseline of LDH levels can serve to predict disease progression within the first year of ICB initiation, but for many patients, the prediction does not considerably precede the detection of progression by imaging scans. Another study reports that an increase in tumor burden of less than 20% from baseline, during 3 months into treatment with pembrolizumab, is associated with longer OS of patients with advanced melanoma [54]. However, we note the difficulty in using early increase in tumor load as a response predictor, as this increase can be detected only a while after the initiation of treatment, when patients may have already experienced disease progression. The ability to predict ICB treatment outcomes before treatment, by use of our suggested personalization algorithm, can be a significant contribution to the currently available methodologies for response evaluation.

Our results show that the Breslow thickness, the baseline tumor burden, and the status of nodular melanoma can serve as markers for TTP prediction under pembrolizumab, when integrated and processed by our personalization algorithm. We found that different values of Breslow thickness and status of nodular melanoma are associated with different rates of tumor growth. Breslow thickness has been known as a prognostic biomarker for melanoma [55, 56], and here we show that it has a predictive power. Using the baseline tumor burden as a potential biomarker is supported by Joseph et al. [57], who analyzed the relationships between baseline tumor burden and overall survival of 583 patients with advanced melanoma under pembrolizumab. In addition, the peripheral blood from patients with advanced melanoma has been analyzed, showing that response to pembrolizumab is associated with the ratio between the baseline tumor burden and the reinvigoration of effector CD8+ T cells [42].

Using a small patient cohort (54 patients) for its training, our personalization algorithm yields moderately accurate predictions. We believe that by increasing the size of the training set we will significantly improve the performance of the regression and classification we employed for identification of the individual model parameters. Yet, considering the limited clinical information and the simple mathematical model implemented at the core of the algorithm, the results are encouraging.

One of the major problems in medical biomathematics is its failure to propose algorithms that can be of aid in the medical practice. Specifically, the two significant hurdles to mathematical models of cancer growth becoming clinically useful, are that in most of the models the required input information cannot be extracted in a straightforward manner from data that are routinely collected in the clinics, and that in most cases, the output information is not instructive for the physician’s decision-making process. Wishing to overcome these shortcomings, we developed our algorithm and tested it using data that are routinely collected in the clinics, namely, the sum of diameters (SOD) or sum of the longest diameters (SLD), as prescribed by the RECIST 1.1. In our case, we could increase the physical and mechanistic realism of the description of tumor growth by asking the radiologists to measure, with little additional effort, more informative tumor size parameters than SOD. But the current standards in the field involve longitudinal measurement of SOD, and as our goal commands, we wish to adjust our tools to the reality in the field, rather than developing an idealized solution.

By the same token, our discretization policy, inevitably, entails loss of information. Treating oncologists do not evaluate the patient’s disease progression status continuously, but rather, every 2–4 months, using the costly imaging technology (CT/MRI). As stated above, our goal was to generate clinically relevant output. For that it would be sufficient to align the prediction of disease progression with the time of its effective substantiation by imaging. For this reason, the resolution of TTP predictions is as coarse as the planned CT/MRI scanning schedule. Still, it would be of a significant help to the doctor to know whether the patient is expected to progress early, or will have moderately long TTP, or a very long TTP, as evaluated by RECIST1.1. The discrete categories of TTP used in this study roughly correspond to these possibilities of response duration.

As one can note, most of the recruited patients are non-progressing (censored). Our approach is to use their longitudinal lesion sizes for model training and validation, so that they have the same weight as the progressing patients in the major part of the work. We then sorted the censored patients as a separate category, checking whether the model had not falsely predicted progression for them during the follow-up period. The alternative way for taking account of censored patients is to construct the survival curves, e.g., by the Kaplan–Meier method, and to use log-rank tests or Cox regression for analysis. The latter methodology would be more suitable if we wished to compare two different populations, and to compare between individuals over the whole patient group.

Model simplicity is a prerequisite for generating a beneficial algorithm, since it requires to evaluate only a small number of personal parameters. A more complex model would entail the evaluation of a relatively large number of clinical measurements in the patients for determining the personal models. It should be borne in mind, also, that our evaluation of disease progression was not required to be more sensitive than that of RECIST 1.1, which takes into account only significant changes in tumor load. Our simple model is well suited for the estimation of similarly rough changes in disease progression.

One of the limitations of the personalization algorithm developed here is that it uses the RECIST 1.1 criteria, which include the appearance of new lesions. This option was not evaluated in our algorithm and we determined disease progression only by the change in size of the target lesions between following imaging scans. Inspecting the clinical patient data, we noted that in about 50% of those in whom new lesions were detected, treatment by pembrolizumab was continued after detection, practically implying that often clinicians do not consider the new lesion criterion as progressive disease. This finding is in line with the recent understanding that formation of new lesions under immunotherapy does not necessarily indicate actual progressive disease [58, 59]. Indeed, in the recently developed immune-related RECIST (irRECIST) criteria, pertinent to immunotherapy, appearance of new lesions is not a criterion for progressive disease [54]. The indicated response is then “unconfirmed progressive disease”, and validation is required in another imaging scan, at least 4 weeks later. Adaptation of our algorithm to the irRECIST criteria will be made upon clinical validation of these criteria as part of the clinical follow-up routine.

Future recommendations for improving the predictive power of our personalization algorithm include training by a larger dataset, as well as validation of the algorithm by clinical data from an independent dataset. Following improvements in the prediction accuracy, our algorithm can be used as a tool in selecting personal treatment. In addition, our innovative methodology can be adapted to other available immunotherapies, including anti-CTLA-4, anti-PD-1 combination, or other immunotherapies when becoming clinically available. Taken together, this study demonstrates that using computational algorithms for predicting the response to immunotherapy in patients with metastatic melanoma is feasible in the clinical realm.

## Conclusions

Our results suggest that personalization of a mathematical mechanistic model by various clinical and molecular pretreatment measurements, can serve for predicting TTP in the clinical setting. Using the developed algorithm to predict the TTP before immunotherapy application can guide the physician decision-making, save several months of administration of an incompatible drug, and significantly improve the quality of life of the patients. Following validation by a new dataset of pembrolizumab-treated patients with advanced melanoma, our algorithm will serve as a tool in the decision-making process of treating physicians. In the future, our algorithm can be adapted to other available therapies, by adjustment of the mathematical mechanistic model, using pertinent clinical data.

## Data Availability

The datasets used and/or analyzed during the current study are available from the corresponding author on reasonable request.

## Abbreviations

ICB: immune checkpoint blockers
CTLA-4: cytotoxic T-lymphocyte antigen 4
CT: computed tomography
PD-1: programmed cell death 1
PD-L1: programmed death-ligand 1
ADT: androgen deprivation therapy
TTP: time to disease progression
APCs: antigen-presenting cells
TILs: effector CD8+ tumor infiltrating lymphocytes
HMC: Hadassah Medical Center
UMM: University Medical Center Mannheim
PET-CT: Positron Emission Tomography CT
MRI: magnetic resonance imaging
RECIST 1.1: Response Evaluation Criteria In Solid Tumors
SOD: sum of diameters
LDH: lactate dehydrogenase
LY%: relative counts of blood lymphocytes
ROC: receiver operating characteristic
k-NN: K-Nearest Neighbors
LOO CV: Leave-One-Out cross validation
OS: overall survival
irRECIST: immune-related RECIST

## References

[1] Siegel RL, Miller KD, Jemal A (2018). Cancer statistics, 2018. CA Cancer J Clin.

[2] Friedman RJ, Rigel DS, Kopf AW (1985). Early detection of malignant melanoma: the role of physician examination and self-examination of the skin. CA Cancer J Clin.

[3] Terushkin V, Halpern AC (2009). Melanoma early detection. Hematol/Oncol Clin.

[4] Schadendorf D, van Akkooi AC, Berking C, Griewank KG, Gutzmer R, Hauschild A, Stang A, Roesch A, Ugurel S (2018). Melanoma. Lancet.

[5] Ott PA, Hodi FS, Robert C (2013). CTLA-4 and PD-1/PD-L1 blockade: new immunotherapeutic modalities with durable clinical benefit in melanoma patients. Clin Cancer Res.

[6] Pardoll DM (2012). The blockade of immune checkpoints in cancer immunotherapy. Nat Rev Cancer.

[7] Hodi FS, O’Day SJ, McDermott DF, Weber RW, Sosman JA, Haanen JB, Gonzalez R, Robert C, Schadendorf D, Hassel JC (2010). Improved survival with ipilimumab in patients with metastatic melanoma. N Engl J Med.

[8] Wolchok JD, Neyns B, Linette G, Negrier S, Lutzky J, Thomas L, Waterfield W, Schadendorf D, Smylie M, Guthrie T (2010). Ipilimumab monotherapy in patients with pretreated advanced melanoma: a randomised, double-blind, multicentre, phase 2, dose-ranging study. Lancet Oncol.

[9] Robert C, Schachter J, Long GV, Arance A, Grob JJ, Mortier L, Daud A, Carlino MS, McNeil C, Lotem M (2015). Pembrolizumab versus ipilimumab in advanced melanoma. N Engl J Med.

[10] Schachter J, Ribas A, Long GV, Arance A, Grob J-J, Mortier L, Daud A, Carlino MS, McNeil C, Lotem M (2017). Pembrolizumab versus ipilimumab for advanced melanoma: final overall survival results of a multicentre, randomised, open-label phase 3 study (KEYNOTE-006). Lancet.

[11] Larkin J, Chiarion-Sileni V, Gonzalez R, Grob JJ, Cowey CL, Lao CD, Schadendorf D, Dummer R, Smylie M, Rutkowski P (2015). Combined nivolumab and ipilimumab or monotherapy in untreated melanoma. N Engl J Med.

[12] Wolchok JD, Chiarion-Sileni V, Gonzalez R, Rutkowski P, Grob J-J, Cowey CL, Lao CD, Wagstaff J, Schadendorf D, Ferrucci PF (2017). Overall survival with combined nivolumab and ipilimumab in advanced melanoma. N Engl J Med.

[13] Robert C, Ribas A, Hamid O, Daud A, Wolchok JD, Joshua AM, Hwu W-J, Weber JS, Gangadhar TC, Joseph RW (2017). Durable complete response after discontinuation of pembrolizumab in patients with metastatic melanoma. J Clin Oncol.

[14] Wang Q, Gao J, Wu X (2018). Pseudoprogression and hyperprogression after checkpoint blockade. Int Immunopharmacol.

[15] Fusi A, Festino L, Botti G, Masucci G, Melero I, Lorigan P, Ascierto PA (2015). PD-L1 expression as a potential predictive biomarker. Lancet Oncol.

[16] Sunshine J, Taube JM (2015). PD-1/PD-L1 inhibitors. Curr Opin Pharmacol.

[17] Weide B, Martens A, Hassel JC, Berking C, Postow MA, Bisschop K, Simeone E, Mangana J, Schilling B, Di Giacomo A-M (2016). Baseline biomarkers for outcome of melanoma patients treated with pembrolizumab. Clin Cancer Res.

[18] Nosrati A, Tsai KK, Goldinger SM, Tumeh P, Grimes B, Loo K, Algazi AP, Nguyen-Kim TDL, Levesque M, Dummer R (2017). Evaluation of clinicopathological factors in PD-1 response: derivation and validation of a prediction scale for response to PD-1 monotherapy. Br J Cancer.

[19] Dronca RS, Liu X, Harrington SM, Chen L, Cao S, Kottschade LA, McWilliams RR, Block MS, Nevala WK, Thompson MA (2016). T cell Bim levels reflect responses to anti-PD-1 cancer therapy. JCI Insight.

[20] Chen P-L, Roh W, Reuben A, Cooper ZA, Spencer CN, Prieto PA, Miller JP, Bassett RL, Gopalakrishnan V, Wani K (2016). Analysis of immune signatures in longitudinal tumor samples yields insight into biomarkers of response and mechanisms of resistance to immune checkpoint blockade. Cancer Discov.

[21] Jacquelot N, Roberti M, Enot D, Rusakiewicz S, Ternès N, Jegou S, Woods D, Sodré A, Hansen M, Meirow Y (2017). Predictors of responses to immune checkpoint blockade in advanced melanoma. Nat Commun.

[22] Agur Z, Halevi-Tobias K, Kogan Y, Shlagman O (2016). Employing dynamical computational models for personalizing cancer immunotherapy. Expert Opin Biol Ther.

[23] Kogan Y, Halevi-Tobias K, Elishmereni M, Vuk-Pavlović S, Agur Z (2012). Reconsidering the paradigm of cancer immunotherapy by computationally aided real-time personalization. Cancer Res.

[24] Elishmereni M, Kheifetz Y, Shukrun I, Bevan GH, Nandy D, McKenzie KM, Kohli M, Agur Z (2016). Predicting time to castration resistance in hormone sensitive prostate cancer by a personalization algorithm based on a mechanistic model integrating patient data. Prostate.

[25] Agur Z, Vuk-Pavlovic S (2012). Mathematical modeling in immunotherapy of cancer: personalizing clinical trials. Mol Ther.

[26] Agur Z, Vuk-Pavlovic S (2012). Personalizing immunotherapy: balancing predictability and precision. Oncoimmunology.

[27] Barrio MM, Abes R, Colombo M, Pizzurro G, Boix C, Roberti MP, Gelize E, Rodriguez-Zubieta M, Mordoh J, Teillaud J-L (2012). Human macrophages and dendritic cells can equally present MART-1 antigen to CD8+ T cells after phagocytosis of gamma-irradiated melanoma cells. PLoS ONE.

[28] Von Euw EM, Barrio MM, Furman D, Bianchini M, Levy EM, Yee C, Li Y, Wainstok R, Mordoh J (2007). Monocyte-derived dendritic cells loaded with a mixture of apoptotic/necrotic melanoma cells efficiently cross-present gp100 and MART-1 antigens to specific CD8+ T lymphocytes. J Transl Med.

[29] Lee T-H, Cho Y-H, Lee M-G (2007). Larger numbers of immature dendritic cells augment an anti-tumor effect against established murine melanoma cells. Biotechnol Lett.

[30] de Pillis L, Gallegos A, Radunskaya A (2013). A model of dendritic cell therapy for melanoma. Front Oncol.

[31] Ludewig B, Krebs P, Junt T, Metters H, Ford NJ, Anderson RM, Bocharov G (2004). Determining control parameters for dendritic cell-cytotoxic T lymphocyte interaction. Eur J Immunol.

[32] Bossi G, Gerry AB, Paston SJ, Sutton DH, Hassan NJ, Jakobsen BK (2013). Examining the presentation of tumor-associated antigens on peptide-pulsed T2 cells. Oncoimmunology.

[33] Taylor GP, Hall SE, Navarrete S, Michie CA, Davis R, Witkover AD, Rossor M, Nowak MA, Rudge P, Matutes E (1999). Effect of lamivudine on human T-cell leukemia virus type 1 (HTLV-1) DNA copy number, T-cell phenotype, and anti-tax cytotoxic T-cell frequency in patients with HTLV-1-associated myelopathy. J Virol.

[34] Carlson JA (2003). Tumor doubling time of cutaneous melanoma and its metastasis. Am J Dermatopathol.

[35] Kuznetsov VA (1991). A mathematical model for the interaction between cytotoxic T lymphocytes and tumour cells. Analysis of the growth, stabilization, and regression of a B-cell lymphoma in mice chimeric with respect to the major histocompatibility complex. Biomed Sci.

[36] Kuznetsov VA, Makalkin IA, Taylor MA, Perelson AS (1994). Nonlinear dynamics of immunogenic tumors: parameter estimation and global bifurcation analysis. Bull Math Biol.

[37] Kuznetsov VA, Zhivoglyadov VP, Stepanova LA (1993). Kinetic approach and estimation of the parameters of cellular interaction between the immunity system and a tumor. Arch Immunol Ther Exp (Warsz).

[38] Kronik N, Kogan Y, Elishmereni M, Halevi-Tobias K, Vuk-Pavlovic S, Agur Z (2010). Predicting outcomes of prostate cancer immunotherapy by personalized mathematical models. PLoS ONE.

[39] Tsur N, Kogan Y, Rehm M, Agur Z (2019). Response of patients with melanoma to immune checkpoint blockade – insights gleaned from analysis of a new mathematical mechanistic model. J Theor Biol.

[40] Eisenhauer E, Therasse P, Bogaerts J, Schwartz L, Sargent D, Ford R, Dancey J, Arbuck S, Gwyther S, Mooney M (2009). New response evaluation criteria in solid tumours: revised RECIST guideline (version 1.1). Eur J Cancer.

[41] Joseph WL, Morton DL, Adkins PC (1971). Variation in tumor doubling time in patients with pulmonary metastatic disease. J Surg Oncol.

[42] Huang AC, Postow MA, Orlowski RJ, Mick R, Bengsch B, Manne S, Xu W, Harmon S, Giles JR, Wenz B (2017). T-cell invigoration to tumour burden ratio associated with anti-PD-1 response. Nature.

[43] Warrens MJ (2013). A comparison of Cohen’s kappa and agreement coefficients by Corrado Gini. Int J.

[44] Fujii T, Naing A, Rolfo C, Hajjar J (2018). Biomarkers of response to immune checkpoint blockade in cancer treatment. Crit Rev Oncol/Hematol.

[45] Sharma P, Allison JP (2015). The future of immune checkpoint therapy. Science.

[46] Garrido MJ, Berraondo P, Trocóniz IF (2016). Commentary on pharmacometrics for immunotherapy.

[47] Nishino M, Ramaiya NH, Hatabu H, Hodi FS (2017). Monitoring immune-checkpoint blockade: response evaluation and biomarker development. Nat Rev Clin Oncol.

[48] Kohn CG, Zeichner SB, Chen Q, Montero AJ, Goldstein DA, Flowers CR (2017). Cost-effectiveness of immune checkpoint inhibition in BRAF wild-type advanced melanoma. J Clin Oncol.

[49] Ribas A, Hamid O, Daud A, Hodi FS, Wolchok JD, Kefford R, Joshua AM, Patnaik A, Hwu W-J, Weber JS (2016). Association of pembrolizumab with tumor response and survival among patients with advanced melanoma. JAMA.

[50] Robert C, Ribas A, Wolchok JD, Hodi FS, Hamid O, Kefford R, Weber JS, Joshua AM, Hwu W-J, Gangadhar TC (2014). Anti-programmed-death-receptor-1 treatment with pembrolizumab in ipilimumab-refractory advanced melanoma: a randomised dose-comparison cohort of a phase 1 trial. Lancet.

[51] Ribas A, Puzanov I, Dummer R, Schadendorf D, Hamid O, Robert C, Hodi FS, Schachter J, Pavlick AC, Lewis KD (2015). Pembrolizumab versus investigator-choice chemotherapy for ipilimumab-refractory melanoma (KEYNOTE-002): a randomised, controlled, phase 2 trial. Lancet Oncol.

[52] Kitano S, Nakayama T, Yamashita M (2018). Biomarkers for immune checkpoint inhibitors in malignant melanoma. Front Oncol.

[53] Diem S, Kasenda B, Spain L, Martin-Liberal J, Marconcini R, Gore M, Larkin J (2016). Serum lactate dehydrogenase as an early marker for outcome in patients treated with anti-PD-1 therapy in metastatic melanoma. Br J Cancer.

[54] Nishino M, Giobbie-Hurder A, Manos MP, Bailey N, Buchbinder EI, Ott PA, Ramaiya NH, Hodi FS (2017). Immune-related tumor response dynamics in melanoma patients treated with pembrolizumab: identifying markers for clinical outcome and treatment decisions. Clin Cancer Res.

[55] Breslow A (1970). Thickness, cross-sectional areas and depth of invasion in the prognosis of cutaneous melanoma. Ann Surg.

[56] Morton DL, Davtyan DG, Wanek LA, Foshag LJ, Cochran AJ (1993). Multivariate analysis of the relationship between survival and the microstage of primary melanoma by Clark level and Breslow thickness. Cancer.

[57] Joseph RW, Elassaiss-Schaap J, Kefford R, Hwu WJ, Wolchok JD, Joshua AM, Ribas A, Hodi FS, Hamid O, Robert C, Daud A, Dronca R, Hersey P, Weber JS, Patnaik A, de Alwis DP, Perrone A, Zhang J, Kang SP, Ebbinghaus S, Anderson KM, Gangadhar TC (2018). Baseline tumor size is an independent prognostic factor for overall survival in patients with melanoma treated with pembrolizumab. Clin Cancer Res..

[58] Wolchok JD, Hoos A, O’Day S, Weber JS, Hamid O, Lebbé C, Maio M, Binder M, Bohnsack O, Nichol G (2009). Guidelines for the evaluation of immune therapy activity in solid tumors: immune-related response criteria. Clin Cancer Res.

[59] Hodi FS, Hwu W-J, Kefford R, Weber JS, Daud A, Hamid O, Patnaik A, Ribas A, Robert C, Gangadhar TC (2016). Evaluation of immune-related response criteria and RECIST v1. 1 in patients with advanced melanoma treated with pembrolizumab. J Clin Oncol.

